# 3-(1*H*-Benzotriazol-1-yl)-1-(3-methoxy­phen­yl)propan-1-one

**DOI:** 10.1107/S1600536809004127

**Published:** 2009-02-11

**Authors:** Guang-Jiu Li, Kong-Cheng Hu

**Affiliations:** a College of Life Science and Pharmaceutical Engineering, Nanjing University of Technology, 210009 Nanjing, Jiangsu, People’s Republic of China

## Abstract

In the title mol­ecule, C_16_H_15_N_3_O_2_, the benzotriazole fragment and the benzene ring form a dihedral angle of 75.02 (1)°. In the crystal structure, mol­ecules related by translation along the *a* axis are linked into chains *via* weak C—H⋯π inter­actions.

## Related literature

For the pharmacological activity of 1*H*-benzotriazole derivatives, see: Chen & Wu (2005 ▶). Some details of the synthesis have been described by Zhu *et al.* (2007 ▶). For reference values of geometric parameters in organic mol­ecules, see: Allen *et al.* (1987 ▶).
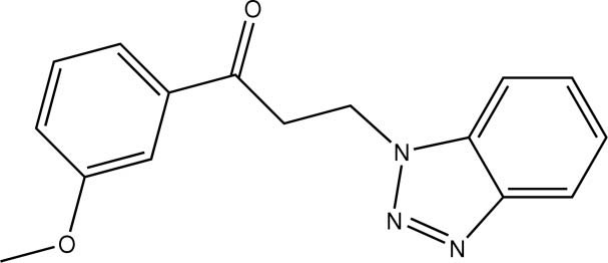


## Experimental

### 

#### Crystal data

C_16_H_15_N_3_O_2_
*M* *_r_* = 281.31
Monoclinic, 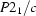

*a* = 5.3583 (14) Å
*b* = 12.976 (4) Å
*c* = 19.688 (5) Å
β = 91.146 (4)°
*V* = 1368.6 (6) Å^3^
*Z* = 4
Mo *K*α radiation
μ = 0.09 mm^−1^
*T* = 293 (2) K
0.21 × 0.15 × 0.07 mm

#### Data collection

Siemens SMART 1000 CCD area-detector diffractometer
Absorption correction: multi-scan (*SADABS*; Sheldrick, 1996 ▶) *T*_min_ = 0.981, *T*_max_ = 0.994
7461 measured reflections
2693 independent reflections
2279 reflections with *I* > 2σ(*I*)
*R*_int_ = 0.018

#### Refinement

*R*[*F* ^2^ > 2σ(*F* ^2^)] = 0.041
*wR*(*F* ^2^) = 0.112
*S* = 1.04
2693 reflections
190 parameters
H-atom parameters constrained
Δρ_max_ = 0.14 e Å^−3^
Δρ_min_ = −0.23 e Å^−3^

### 

Data collection: *SMART* (Siemens, 1996 ▶); cell refinement: *SAINT* (Siemens, 1996 ▶); data reduction: *SAINT*; program(s) used to solve structure: *SHELXS97* (Sheldrick, 2008 ▶); program(s) used to refine structure: *SHELXL97* (Sheldrick, 2008 ▶); molecular graphics: *SHELXTL* (Sheldrick, 2008 ▶); software used to prepare material for publication: *SHELXTL*, *PARST* (Nardelli, 1995 ▶) and *PLATON* (Spek, 2003 ▶).

## Supplementary Material

Crystal structure: contains datablocks global, I. DOI: 10.1107/S1600536809004127/cv2510sup1.cif

Structure factors: contains datablocks I. DOI: 10.1107/S1600536809004127/cv2510Isup2.hkl

Additional supplementary materials:  crystallographic information; 3D view; checkCIF report

## Figures and Tables

**Table 1:** Hydrogen-bond geometry (Å, °)

*D*—H⋯*A*	*D*—H	H⋯*A*	*D*⋯*A*	*D*—H⋯*A*
C9—H9*A*⋯*Cg*1^i^	0.97	2.74	3.504	136

Symmetry code: (i) 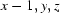. *Cg*1 is the centroid of atoms C10–C15.

**Fig. 1.**
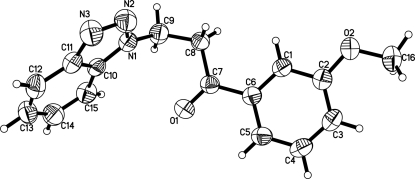
The molecular structure of (I) showing 50% probability displacement ellipsoids and the atom numbering scheme.

## supplementary crystallographic information

### Comment

1*H*-Benzotriazole and its derivatives exhibit a broad spectrum of pharmacological activities, such as antifungal, antitumor and antineoplastic (Chen & Wu, 2005). In order to search for new benzotriazole derivatives with higher bioactivity, the title compound, (I), was synthesized and its structure is shown here.

In the title molecule (Fig. 1), all bond lengths and angles are within normal ranges (Allen *et al.*, 1987). The benzotriazole system is almost planar with a dihedral angle of 1.45 (1)° between the triazole (N1–N3/C10/C11) and benzene (C10—C15) rings. The whole molecular is non-planar with a dihedral angle of 75.02 (1)° between the benzotriazole fragment  and benzene C1–C6 ring. In the crystal, the molecules related by translation along axis *a* are linked into chains *via* the weak C—H···π interactions (Table 1).

### Experimental

The title compound was prepared according to the literature method of Zhu *et al.*(2007). Single crystals suitable for X-ray diffraction were obtained by slow evaporation of an ethyl acetate solution at room temperature over a period of one week.

### Refinement

All H atoms were located in difference Fourier maps and constrained to ride on their parent atoms, with C—H distances in the range 0.93–0.97 Å, and with *U*_iso_(H) = 1.2 *U*_eq_(C) and 1.5 *U*_eq_(methyl C).

### Crystal data

**Table:**

C_16_H_15_N_3_O_2_	*F*(000) = 592
*M**_r_* = 281.31	*D*_x_ = 1.365 Mg m^−^^3^
Monoclinic, *P*2_1_/*c*	Mo *K*α radiation, λ = 0.71073 Å
*a* = 5.3583 (14) Å	Cell parameters from 3062 reflections
*b* = 12.976 (4) Å	θ = 2.6–25.9°
*c* = 19.688 (5) Å	µ = 0.09 mm^−^^1^
β = 91.146 (4)°	*T* = 293 K
*V* = 1368.6 (6) Å^3^	Block, colourless
*Z* = 4	0.21 × 0.15 × 0.07 mm

### Data collection

**Table:**

Siemens SMART 1000 CCD area-detector diffractometer	2693 independent reflections
Radiation source: fine-focus sealed tube	2279 reflections with *I* > 2σ(*I*)
graphite	*R*_int_ = 0.018
Detector resolution: 8.33 pixels mm^-1^	θ_max_ = 26.0°, θ_min_ = 1.9°
ω scans	*h* = −6→6
Absorption correction: multi-scan (*SADABS*; Sheldrick, 1996)	*k* = −16→16
*T*_min_ = 0.981, *T*_max_ = 0.994	*l* = −24→17
7461 measured reflections	

### Refinement

**Table:**

Refinement on *F*^2^	Primary atom site location: structure-invariant direct methods
Least-squares matrix: full	Secondary atom site location: difference Fourier map
*R*[*F*^2^ > 2σ(*F*^2^)] = 0.041	Hydrogen site location: inferred from neighbouring sites
*wR*(*F*^2^) = 0.112	H-atom parameters constrained
*S* = 1.04	*w* = 1/[σ^2^(*F*_o_^2^) + (0.0577*P*)^2^ + 0.249*P*] where *P* = (*F*_o_^2^ + 2*F*_c_^2^)/3
2693 reflections	(Δ/σ)_max_ < 0.001
190 parameters	Δρ_max_ = 0.14 e Å^−^^3^
0 restraints	Δρ_min_ = −0.23 e Å^−^^3^

### Special details

**Table:**

Geometry. All e.s.d.'s (except the e.s.d. in the dihedral angle between two l.s. planes) are estimated using the full covariance matrix. The cell e.s.d.'s are taken into account individually in the estimation of e.s.d.'s in distances, angles and torsion angles; correlations between e.s.d.'s in cell parameters are only used when they are defined by crystal symmetry. An approximate (isotropic) treatment of cell e.s.d.'s is used for estimating e.s.d.'s involving l.s. planes.
Refinement. Refinement of *F*^2^ against ALL reflections. The weighted *R*-factor *wR* and goodness of fit *S* are based on *F*^2^, conventional *R*-factors *R* are based on *F*, with *F* set to zero for negative *F*^2^. The threshold expression of *F*^2^ > σ(*F*^2^) is used only for calculating *R*-factors(gt) *etc*. and is not relevant to the choice of reflections for refinement. *R*-factors based on *F*^2^ are statistically about twice as large as those based on *F*, and *R*- factors based on ALL data will be even larger.

### Fractional atomic coordinates and isotropic or equivalent isotropic displacement parameters (Å^2^)

**Table:**

	*x*	*y*	*z*	*U*_iso_*/*U*_eq_	
O1	0.2340 (2)	0.48618 (9)	0.09618 (6)	0.0607 (3)	
O2	−0.2250 (2)	0.87656 (8)	−0.00231 (6)	0.0570 (3)	
N1	0.0340 (2)	0.44726 (9)	0.23515 (6)	0.0427 (3)	
C6	0.1036 (2)	0.64278 (10)	0.04534 (6)	0.0396 (3)	
C2	−0.0452 (3)	0.80221 (11)	−0.00439 (7)	0.0444 (3)	
C1	−0.0680 (3)	0.72307 (11)	0.04300 (7)	0.0428 (3)	
H1A	−0.1990	0.7240	0.0733	0.051*	
C10	0.2033 (2)	0.37083 (10)	0.24527 (7)	0.0390 (3)	
C7	0.0797 (2)	0.55465 (11)	0.09372 (7)	0.0415 (3)	
C5	0.3012 (3)	0.64292 (12)	0.00019 (7)	0.0462 (3)	
H5A	0.4176	0.5898	0.0012	0.055*	
C8	−0.1429 (3)	0.55249 (11)	0.13963 (7)	0.0458 (3)	
H8A	−0.2938	0.5586	0.1119	0.055*	
H8B	−0.1348	0.6120	0.1694	0.055*	
N2	0.0569 (3)	0.51863 (10)	0.28486 (7)	0.0573 (4)	
C12	0.5305 (3)	0.33871 (12)	0.32934 (8)	0.0541 (4)	
H12A	0.6189	0.3565	0.3687	0.065*	
C13	0.5867 (3)	0.25211 (13)	0.29353 (8)	0.0579 (4)	
H13A	0.7178	0.2106	0.3087	0.070*	
N3	0.2374 (3)	0.49148 (10)	0.32671 (7)	0.0589 (4)	
C3	0.1519 (3)	0.80179 (12)	−0.04920 (7)	0.0498 (4)	
H3B	0.1684	0.8543	−0.0810	0.060*	
C11	0.3340 (3)	0.39961 (11)	0.30415 (7)	0.0440 (3)	
C15	0.2589 (3)	0.28109 (11)	0.20939 (8)	0.0505 (4)	
H15A	0.1686	0.2617	0.1706	0.061*	
C4	0.3230 (3)	0.72214 (13)	−0.04583 (7)	0.0518 (4)	
H4A	0.4562	0.7221	−0.0754	0.062*	
C9	−0.1613 (3)	0.45603 (12)	0.18278 (8)	0.0516 (4)	
H9A	−0.3224	0.4553	0.2044	0.062*	
H9B	−0.1536	0.3962	0.1533	0.062*	
C14	0.4524 (3)	0.22363 (12)	0.23434 (9)	0.0590 (4)	
H14A	0.4972	0.1638	0.2116	0.071*	
C16	−0.2412 (4)	0.94720 (13)	−0.05808 (9)	0.0621 (4)	
H16A	−0.3743	0.9952	−0.0507	0.093*	
H16B	−0.2738	0.9098	−0.0994	0.093*	
H16C	−0.0865	0.9839	−0.0616	0.093*	

### Atomic displacement parameters (Å^2^)

**Table:**

	*U*^11^	*U*^22^	*U*^33^	*U*^12^	*U*^13^	*U*^23^
O1	0.0544 (6)	0.0619 (7)	0.0661 (7)	0.0170 (5)	0.0131 (5)	0.0130 (5)
O2	0.0617 (7)	0.0522 (6)	0.0572 (7)	0.0079 (5)	0.0049 (5)	0.0124 (5)
N1	0.0441 (6)	0.0438 (6)	0.0404 (6)	−0.0021 (5)	0.0016 (5)	0.0031 (5)
C6	0.0367 (7)	0.0471 (7)	0.0348 (7)	−0.0026 (6)	−0.0028 (5)	−0.0029 (5)
C2	0.0446 (8)	0.0454 (8)	0.0430 (8)	−0.0040 (6)	−0.0049 (6)	0.0004 (6)
C1	0.0406 (7)	0.0493 (8)	0.0386 (7)	−0.0021 (6)	0.0024 (6)	0.0005 (6)
C10	0.0389 (7)	0.0402 (7)	0.0381 (7)	−0.0062 (5)	0.0051 (5)	0.0047 (5)
C7	0.0368 (7)	0.0471 (8)	0.0405 (7)	0.0005 (6)	−0.0024 (5)	−0.0024 (6)
C5	0.0394 (7)	0.0570 (8)	0.0423 (8)	0.0010 (6)	0.0016 (6)	−0.0028 (6)
C8	0.0358 (7)	0.0545 (8)	0.0471 (8)	0.0020 (6)	0.0005 (6)	0.0077 (6)
N2	0.0683 (9)	0.0498 (7)	0.0537 (8)	0.0062 (6)	0.0001 (7)	−0.0052 (6)
C12	0.0559 (9)	0.0585 (9)	0.0476 (9)	−0.0050 (7)	−0.0089 (7)	0.0084 (7)
C13	0.0536 (9)	0.0557 (9)	0.0644 (10)	0.0074 (7)	−0.0007 (8)	0.0161 (8)
N3	0.0747 (9)	0.0522 (8)	0.0493 (8)	0.0034 (7)	−0.0080 (7)	−0.0087 (6)
C3	0.0522 (9)	0.0562 (9)	0.0409 (8)	−0.0107 (7)	−0.0002 (6)	0.0070 (6)
C11	0.0497 (8)	0.0437 (7)	0.0387 (7)	−0.0074 (6)	0.0006 (6)	0.0021 (6)
C15	0.0588 (9)	0.0473 (8)	0.0453 (8)	−0.0034 (7)	−0.0015 (7)	−0.0053 (6)
C4	0.0453 (8)	0.0683 (10)	0.0420 (8)	−0.0070 (7)	0.0079 (6)	0.0004 (7)
C9	0.0385 (8)	0.0610 (9)	0.0552 (9)	−0.0070 (7)	−0.0021 (6)	0.0119 (7)
C14	0.0703 (11)	0.0457 (8)	0.0613 (10)	0.0074 (8)	0.0072 (8)	−0.0018 (7)
C16	0.0732 (11)	0.0537 (9)	0.0591 (10)	0.0047 (8)	−0.0062 (8)	0.0119 (8)

### Geometric parameters (Å, °)

**Table:**

O1—C7	1.2137 (17)	C8—H8B	0.9700
O2—C2	1.3649 (18)	N2—N3	1.3063 (19)
O2—C16	1.4316 (18)	C12—C13	1.363 (2)
N1—N2	1.3511 (17)	C12—C11	1.399 (2)
N1—C10	1.3563 (17)	C12—H12A	0.9300
N1—C9	1.4586 (18)	C13—C14	1.407 (2)
C6—C1	1.3896 (19)	C13—H13A	0.9300
C6—C5	1.3961 (19)	N3—C11	1.3769 (19)
C6—C7	1.4954 (19)	C3—C4	1.382 (2)
C2—C3	1.389 (2)	C3—H3B	0.9300
C2—C1	1.394 (2)	C15—C14	1.361 (2)
C1—H1A	0.9300	C15—H15A	0.9300
C10—C11	1.3933 (19)	C4—H4A	0.9300
C10—C15	1.397 (2)	C9—H9A	0.9700
C7—C8	1.511 (2)	C9—H9B	0.9700
C5—C4	1.377 (2)	C14—H14A	0.9300
C5—H5A	0.9300	C16—H16A	0.9600
C8—C9	1.517 (2)	C16—H16B	0.9600
C8—H8A	0.9700	C16—H16C	0.9600
			
C2—O2—C16	117.48 (12)	C12—C13—C14	122.05 (15)
N2—N1—C10	110.12 (12)	C12—C13—H13A	119.0
N2—N1—C9	120.80 (12)	C14—C13—H13A	119.0
C10—N1—C9	128.99 (12)	N2—N3—C11	107.97 (12)
C1—C6—C5	119.18 (13)	C4—C3—C2	118.97 (13)
C1—C6—C7	121.97 (12)	C4—C3—H3B	120.5
C5—C6—C7	118.83 (12)	C2—C3—H3B	120.5
O2—C2—C3	124.61 (13)	N3—C11—C10	108.32 (13)
O2—C2—C1	115.41 (13)	N3—C11—C12	131.29 (14)
C3—C2—C1	119.98 (14)	C10—C11—C12	120.37 (14)
C6—C1—C2	120.50 (13)	C14—C15—C10	116.26 (14)
C6—C1—H1A	119.7	C14—C15—H15A	121.9
C2—C1—H1A	119.7	C10—C15—H15A	121.9
N1—C10—C11	104.48 (12)	C5—C4—C3	121.66 (14)
N1—C10—C15	133.14 (13)	C5—C4—H4A	119.2
C11—C10—C15	122.38 (13)	C3—C4—H4A	119.2
O1—C7—C6	121.22 (12)	N1—C9—C8	113.99 (12)
O1—C7—C8	120.51 (13)	N1—C9—H9A	108.8
C6—C7—C8	118.28 (12)	C8—C9—H9A	108.8
C4—C5—C6	119.69 (14)	N1—C9—H9B	108.8
C4—C5—H5A	120.2	C8—C9—H9B	108.8
C6—C5—H5A	120.2	H9A—C9—H9B	107.7
C7—C8—C9	114.27 (12)	C15—C14—C13	121.90 (15)
C7—C8—H8A	108.7	C15—C14—H14A	119.0
C9—C8—H8A	108.7	C13—C14—H14A	119.0
C7—C8—H8B	108.7	O2—C16—H16A	109.5
C9—C8—H8B	108.7	O2—C16—H16B	109.5
H8A—C8—H8B	107.6	H16A—C16—H16B	109.5
N3—N2—N1	109.11 (12)	O2—C16—H16C	109.5
C13—C12—C11	117.02 (15)	H16A—C16—H16C	109.5
C13—C12—H12A	121.5	H16B—C16—H16C	109.5
C11—C12—H12A	121.5		
			
C16—O2—C2—C3	13.0 (2)	N1—N2—N3—C11	−0.44 (17)
C16—O2—C2—C1	−167.35 (13)	O2—C2—C3—C4	179.60 (13)
C5—C6—C1—C2	0.8 (2)	C1—C2—C3—C4	0.0 (2)
C7—C6—C1—C2	−177.70 (12)	N2—N3—C11—C10	0.20 (17)
O2—C2—C1—C6	179.60 (12)	N2—N3—C11—C12	178.69 (15)
C3—C2—C1—C6	−0.8 (2)	N1—C10—C11—N3	0.12 (15)
N2—N1—C10—C11	−0.40 (14)	C15—C10—C11—N3	179.28 (13)
C9—N1—C10—C11	−176.92 (12)	N1—C10—C11—C12	−178.56 (12)
N2—N1—C10—C15	−179.42 (15)	C15—C10—C11—C12	0.6 (2)
C9—N1—C10—C15	4.1 (2)	C13—C12—C11—N3	−177.86 (15)
C1—C6—C7—O1	−178.41 (13)	C13—C12—C11—C10	0.5 (2)
C5—C6—C7—O1	3.1 (2)	N1—C10—C15—C14	177.59 (14)
C1—C6—C7—C8	1.83 (19)	C11—C10—C15—C14	−1.3 (2)
C5—C6—C7—C8	−176.67 (12)	C6—C5—C4—C3	−0.7 (2)
C1—C6—C5—C4	−0.1 (2)	C2—C3—C4—C5	0.7 (2)
C7—C6—C5—C4	178.46 (13)	N2—N1—C9—C8	61.53 (17)
O1—C7—C8—C9	−4.4 (2)	C10—N1—C9—C8	−122.27 (15)
C6—C7—C8—C9	175.40 (12)	C7—C8—C9—N1	68.40 (17)
C10—N1—N2—N3	0.54 (16)	C10—C15—C14—C13	1.0 (2)
C9—N1—N2—N3	177.39 (12)	C12—C13—C14—C15	0.1 (3)
C11—C12—C13—C14	−0.8 (2)		

### Hydrogen-bond geometry (Å, °)

**Table:**

*D*—H···*A*	*D*—H	H···*A*	*D*···*A*	*D*—H···*A*
C9—H9A···Cg1^i^	0.97	2.74	3.504	136

Symmetry codes: (i) *x*−1, *y*, *z*.
